# miR-1929-3p Overexpression Alleviates Murine Cytomegalovirus-Induced Hypertensive Myocardial Remodeling by Suppressing Ednra/NLRP3 Inflammasome Activation

**DOI:** 10.1155/2020/6653819

**Published:** 2020-12-30

**Authors:** YongJia Wang, Zhen Huang, Hua Zhong, LaMei Wang, DongMei Xi, YunZhong Shi, Wei Zhou, YongMin Liu, Na Tang, Fang He

**Affiliations:** ^1^Department of Pathophysiology, Key Laboratory of Education, Ministry of Xinjiang Endemic and Ethnic Diseases, Medical College of Shihezi University, Shihezi 832002, China; ^2^Centre of Medical Functional Experiments, Medical College of Shihezi University, Shihezi 832002, China

## Abstract

MicroRNAs (miRNAs) play crucial roles in the development of essential hypertension (EH). Previously, we found that the expression of miR-1929-3p was decreased in C57BL/6 mice with hypertension induced by murine cytomegalovirus (MCMV). In this study, we explored the role of miR-1929-3p in hypertension myocardial remodeling in MCMV-infected mice. First, we measured MCMV DNA and host IgG and IgM after infection and determined the expression of miR-1929-3p and its target gene endothelin A receptor (Ednra) mRNA in the myocardium of mice. Then, we performed invasive blood pressure (BP) monitoring. Heart-to-body weight ratio (HW/BW%), along with mRNA levels of B-type natriuretic peptide (BNP) and beta myosin heavy chain (*β*-MHC), revealed myocardial remodeling. Hematoxylin/eosin and Masson's trichrome staining indicated morphological changes in the myocardium. Cardiac function was assessed via echocardiography. Moreover, MCMV-infected mice were injected with recombinant adeno-associated virus- (rAAV-) miR-1929-3p overexpression vector. Immunohistochemistry and western blotting showed the expression of Ednra and the activation of NOD-like receptor pyrin domain containing 3 (NLRP3) inflammasome. And enzyme-linked immunosorbent assay (ELISA) revealed the concentrations of endothelin-1 (ET-1), interleukin-1*β* (IL-1*β*), and interleukin-18 (IL-18). In this study, we found that decreased expression of miR-1929-3p in MCMV-infected mice induced high BP and further development of myocardial remodeling cardiac function injury through increased expression of Ednra. Strikingly, overexpression of miR-1929-3p ameliorated these pathological changes of the heart. The positive effect was shown to be associated with inhibition of NLRP3 inflammasome activation and decreased expression of key proinflammatory cytokine IL-1*β*. Collectively, these results indicate that miR-1929-3p overexpression may effectively alleviate EH myocardial remodeling by suppressing Ednra/NLRP3 inflammasome activation in MCMV-infected mice.

## 1. Introduction

High blood pressure (BP) is a leading risk factor for global disease burden [1]. Hypertension has been estimated to account for approximately 9.4 million deaths annually. EH constitutes about 90-95% of all hypertension types [2, 3]. The insidious nature of hypertension is underscored by its high prevalence, its mostly asymptomatic nature, and the slow progress in the achievement of treatment targets [4]. Myocardial remodeling is one of the major manifestations of hypertensive heart disease, which is characterized by a change in gene expression leading to molecular, cellular, and interstitial alterations; clinical manifestations include cardiac hypertrophy, fibrosis, and decompensation [5]. Recent studies prompted that EH is regulated by epigenetic mechanisms including viral infection and miRNAs.

HCMV is a ubiquitous *β*-herpesvirus and a member of the cytomegalovirus (CMV) family. It has the largest genome of human herpesviruses (230 kb), encoding a variety of gene products, many of which play an immunomodulatory role in the host [6, 7]. According to a recent survey, the prevalence of HCMV infection is 40%-100% worldwide [8]. Once the host is infected, HCMV cannot be completely eliminated by cell and humoral immunity, resulting in latent infection [9]. Firth et al. discovered that HCMV infection is positively related to systolic blood pressure (SBP) in subjects of a clinical study [10]. Although the complex activity cycle of HCMV involves many regulatory mechanisms and gene products [11, 12], its pathogenic mechanisms remain unclear.

miRNAs are a class of evolutionarily highly conserved single-stranded, noncoding RNAs that negatively regulate the expression of target genes at the posttranscriptional level [13]. Li et al. found that hcmv-miR-UL112 encoded by HCMV is upregulated in hypertensive patients. Then, C57BL/6 mice developed high BP after MCMV infection in their study [14]. Based on these studies, we focused on the role of miRNAs that could participate in the occurrence and development of hypertension.

Hypertension triggers a chronic inflammatory process. Accumulating evidence indicates that the activation of NLRP3 inflammasome is not only involved in the development of EH [15] but is also related to myocardial remodeling [16]. The NLRP3 inflammasome consists of NLRP3, an apoptosis-associated speck-like protein containing a CARD (ASC), and cysteinyl aspartate specific proteinase-1 (caspase-1). NLRP3 is the core protein that activates the NLRP3 inflammasome after recognizing and binding pathogen-associated molecular patterns (PAMP) or danger-associated molecular patterns (DAMP). This activated complex in turn leads to the activation of caspase-1, a proteolytic enzyme that is responsible for the maturation of the downstream proinflammatory cytokines IL-1*β* and IL-18 [17]. miRNAs can affect cardiac function by influencing the activation of NLRP3. For example, miR-223-3p can aggravate myocarditis by inhibiting the expression of the NLRP3 inflammasome [17]. Moreover, miR-135b can reduce the proliferation of cardiomyocytes and restore cardiac function by regulating the NLRP3/caspase-1/IL-1*β* pathway [18].

Recently, our group showed that the host-encoded miR-1929-3p was downregulated in C57BL/6 mice with MCMV-induced hypertension. Bioinformatic prediction and double luciferase validation revealed that Ednra is the target gene of miR-1929-3p [19]. Mechanical stretch induces a cardiac hypertrophic response, partly through the production of ET-1 through Ednra, along with increased IL-18 expression [20]. Based on these studies, we hypothesized that, in MCMV-infected mice, downregulated miR-1929-3p could stimulate the activation of the NLRP3 inflammasome in the heart by suppressing Ednra, which will eventually cause high BP, the development of pathological myocardial remodeling, and cardiac dysfunction. What's more, we explored the possible treatment for alleviating these pathological changes by miR-1929-3p overexpression.

## 2. Materials and Methods

### 2.1. Animals and Ethics Statement

The establishment of the animal model was carried out according to reference 21. We selected 7-month-old C57BL/6 mice (Vital River Laboratory Animal Science and Technology Co., Ltd., Beijing, P.R.C.), approaching middle and old age [22], in which it is easier to induce hypertension and myocardial remodeling. Experimental mice were living in a suitable environment with free feeding, free drinking water, and natural light illumination at a temperature of 18~22°C, with 40%~70% relative humidity and noise < 50 dB. These mice were classified into four groups randomly: control (*n* = 55), MCMV (*n* = 55), MCMV+miR-1929-3p-NC (MCMV+rAAV-miR-1929-3p negative plasmid; *n* = 40), and MCMV+miR-1929-3p (MCMV+rAAV-miR-1929-3p overexpressed plasmid; *n* = 40). Each group was treated with MCMV (Wuhan Institute of Virology, Hubei, China) or NS (normal saline) (1 × 10^5^ pfu/1 mL/week) through intraperitoneal injection for 0 month (7 months old), two months (9 months old), and six months (13 months old) [23]. Then, mouse in the MCMV group were treated with rAAV-miR-1929-3p negative vector or rAAV-miR-1929-3p overexpressed vector (Shanghai Genechem Co., Ltd., China; 1.01 × 10^13^ v.g./mL) through tail vein injection once at 13 months of age. rAAV-miR-1929-3p overexpression vector was established according to instructions. Approval of the protocol was obtained from the Institutional Animal Research Committee of Shihezi Medical University, and Guide for the Care and Use of Laboratory Animals issued by the National Institutes of Health was followed.

### 2.2. MCMV Infection

Detection of the DNA encoding immediately protein 1 (IE1) in the host is evidence of early infection by CMV [24]. CMV-IgM is produced 3-5 days after MCMV infection and lasts for only 12-16 weeks [25]. CMV-IgG is the only antibody that can pass through the placenta, appearing in the host 7-14 days after CMV infection, reaching its peak at 4-8 weeks [26]. CMV-IgM and IgG were credible indicators for CMV infection.

### 2.3. rAAV-miR-1929-3p Overexpression Vector Establishment

The overexpression vector of rAAV-miR-1929-3p was constructed by the AAV Helper-Free System (including virus vector GV412, pAAV-RC vector, and pHelper vector) (GeneChem, Shanghai, China). Firstly, the miR-1929-3p gene was cloned into the AAV-9 virus vector (miRNA-up GV412) to produce the recombinant AAV particles. XL10-Gold competent cells (Stratagene catalog number # 200314) were used for the amplification of recombinant AAV plasmid. Thereafter, the recombinant expression plasmid was cotransfected into AAV-293 cells (providing transacting factors for AAV replication and packaging) with pHelper (carrying adenovirus-derived genes) and pAAV-RC (carrying AAV replication and capsid genes). After 2 to 3 days of transfection, the AAV virus particles released by the infected AAV-293 cells into the supernatant were collected and further concentrated and purified. The genome copy number of the AAV vector was detected by quantitative PCR to determine the virus titer of AAV.

### 2.4. Measurement of BP, HW/BW%, and LVW/BW%

The C57BL/6 mice were anesthetized with sodium pentobarbital (P3761, Sigma-Aldrich, St. Louis, MO, USA) through an intraperitoneal injection (30 mg/kg). An incision was made along the midline of the neck, and the tissues were separated to expose the common carotid artery. Vagus nerves surrounding the carotid artery were separated carefully. A silicone tube (internal diameter: 0.3 mm) was implanted near the artery (diameter: about 0.5 mm) and ligated using 6-0 silk threads. Systolic blood pressure (SBP), diastolic blood pressure (DBP), and mean arterial pressure (MAP) were recorded by invasive blood pressure monitoring. Then, the heart was removed and the left ventricle (LV) was resected to calculate HW/BW% and the LVW/BW%.

### 2.5. Hematoxylin Eosin (H&E) Staining and Masson's Trichrome Staining

Paraffin-embedded sections were stained with Masson's trichrome staining or H&E staining under standard protocols. The cross-sectional area of myocytes and the ratio of collagen area to the total cardiac area were measured or calculated as indexes of cardiac remodeling.

### 2.6. Real-Time Quantitative Reverse Transcription Polymerase Chain Reaction (qRT-PCR)

Total RNA was separated and reversely transcribed to cDNA (Tiangen Biotech, Shanghai, China) based on the manufacturer's instruction. A real-time PCR instrument (ABI 7500, Applied Biosystems, CA, America) was used to amplify the target band, and the PCR reaction system was 20 *μ*L including SYBR Green qPCR Master Mix, forward primers, reverse primers, and cDNA. U6 was considered the internal reference of miR-1929-3p and glyceraldehyde-3-phosphate dehydrogenase (GAPDH) was considered for the other genes.

The sequences of primers (Shanghai Living Creature, China) are as follows [27]:

1. BNP-F: 5′-TCCAGGAGAGACTTCGAAATTC-3′
2. BNP-R: 5′-GCAAGTTTGTGCTGGAAGATAA-3′
3. *β*-MHC-F: 5′-AAGGCCAAGATCGAGACGG-3′
4. *β*-MHC-R: 5′-CCACTTATAGGGGGTTGACGGTG-3′
5. GAPDH-F:5′-TGGCCTTCCGTGTTCCTAC-3′
6. GAPDH-R: 5′-GAGTTGCTGTTGAAGTCGCA-3′
7. Ednra-F: 5′-TCACCGTCTTGAACCTCTGTGC-3′
8. Ednra-R: 5′-GATGGAGACGATTTCAATGGCGG-3′
9. mmu-miR-1929-3p-F: 5′-ACACTCCAGCTGGGCAGCTCATGGAGACCT-3′
10. mmu-miR-1929-3p-R: 5′-TGGTGTCGTGGAGTCG-3′
11. U6-F: 5′-GCTTCGGCAGCACATATACTAAAAT-3′
12. U6-R: 5′-CGCTTCACGAATTTGCGTGTCAT-3′

All gene expression levels were calculated using the 2^−ΔΔCt^ method.

### 2.7. Assessment of Heart Function and Cardiac Structures

We performed anesthesia through intraperitoneal injection with sodium pentobarbital at a dose of 30 mg/kg in live mice. Then, we put the probe on the left side of the chest for M-mode imaging. We record the left ventricular M-mode images with an animal-specific instrument (VisualSonics Vevo 3100, VisualSonics Inc., Toronto, Canada). Left ventricular end-systolic diameter (LVIDs), left ventricular end-diastolic posterior wall thickness (LVPWd), left ventricular end-diastolic diameter (LVIDd), percent fractional shortening (FS%), and percent ejection fraction (EF%) were measured to evaluate the extent of hypertrophy and function of the heart at age of 16 months.

### 2.8. Immunohistochemistry Analysis

After deparaffinization, cardiac sections were subjected to incubation with rabbit primary antibodies anti-NLRP3 (1 : 50; Abcam, Cambridge, UK), ASC (1 : 100; Abcam, Cambridge, UK), and caspase-1 (1 : 100; Abcam, Cambridge, UK) for 24 h at 4°C. Then, the sections were incubated with horseradish peroxidase-conjugated secondary antibody (Invitrogen, Carlsbad, CA, USA) at 37°C for 30 min. Diaminobenzidine/peroxidase substrate was utilized for color development. Integral optical density/myocardial area was employed to analyze the positive staining of tissue sections.

### 2.9. Enzyme-Linked Immunosorbent Assay (ELISA)

Plasma samples were extracted as described above. Enzyme immunoassay (commercially available) was employed to detect ET-1, IL-1*β*, and IL-18 (Elabscience Biotechnology Co. Ltd., Wuhan, China) in plasma and heart tissues. The absorbance was read at 450 nm by a microplate reader (Bio-Rad Laboratories, Hercules, America; Bio-Rad Model 3550 UV).

### 2.10. Western Blotting

After specific treatments, cells were incubated in lysis buffer (PMSF : RIPA = 1 : 100) for 20 min on ice. After centrifugation (12,000 g for 15 min at 4°C), the supernatants were collected and protein concentrations were assessed by the BCA method. After transferring total protein from SDS-PAGE, the PVDF membranes were incubated overnight at 4°C using primary antibodies against NLRP3, ASC, caspase-1, IL-1*β*, IL-18, Ednra (Abcam, Cambridge, UK), and GAPDH (Zsbio Commerce Store, Beijing, China). Band intensity was quantified using Bio-Rad Quantity One software (Bio-Rad) with GAPDH as an internal control.

### 2.11. Statistical Analysis

The SPSS 21.0 statistical software (IBM Corp., Armonk, NY, USA) was utilized for statistical analysis. Measurement data were presented as the mean ± standard deviation. *t*-test was adopted for comparison between two groups. *P* < 0.05 indicated a statistically significant difference.

## 3. Results

### 3.1. MCMV Infection Increases BP and Downregulates miR-1929-3p in C57BL/6 Mice

**Figure 1:**
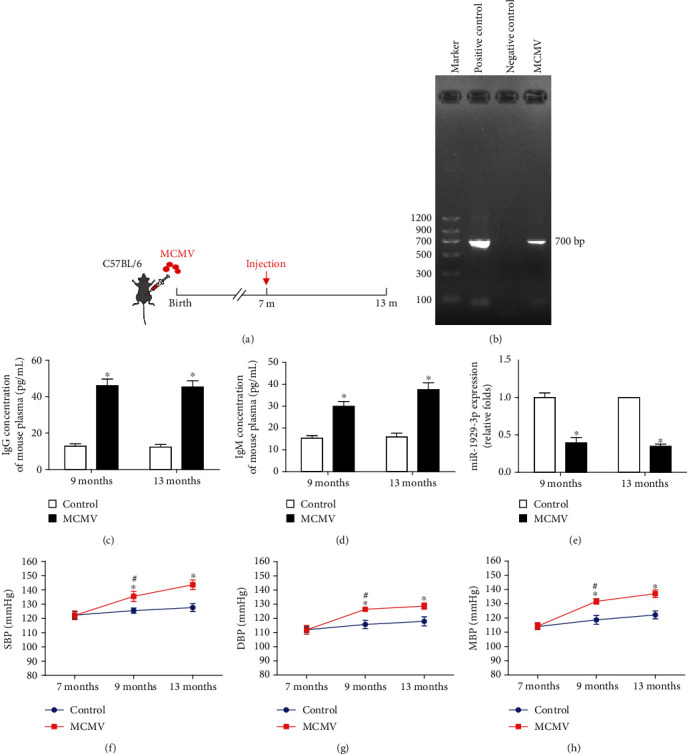
MCMV infection raised BP and decreased the expression of miR-1929-3p in C57BL/6 mice. (a) Schematic of intervention here and in Figure 2 in the experimental group. (b) PCR analysis of the MCMV IE gene in the myocardium of C57BL/6 mice. (c) ELISA detection of IgG concentration in plasma. (d) ELISA detection of IgM concentration in plasma. (e) qRT-PCR analysis of miR-1929-3p expression in the myocardium. The value was normalized to the value observed with the control groups, which was set to 1: (f) SBP; (g) DBP; (h) MAP. The data are expressed as the means ± SEM (*n* = 5), ^∗^*P* < 0.05, the MCMV groups vs. the age-matched control groups. ^#^*P* < 0.05, the MCMV 9-month groups vs. the control 7-month groups.

To confirm successful infection, we detected the expression of MCMV IE DNA by PCR. The IE DNA was clearly expressed in the thoracic aorta of infected mice (Figures 1(a) and 1(b)). To assess toxigenic infection, we measured the concentration of IgG and IgM antibodies in mice by ELISA. Both IgG and IgM surged after infection for 2 months and lasted for at least 4 months (*P* < 0.05, Figures 1(c) and 1(d)). Moreover, the expression of miR-1929-3p in the myocardium of MCMV-infected mice was significantly decreased (*P* < 0.05, Figure 1(e)). After infection for 2 months, the increase of SBP, DBP, and MAP in both groups revealed significantly higher BP (*P* < 0.05), which elevated even further in mice along with extended MCMV infection (*P* < 0.05, Figures 1(f)–1(h)).

### 3.2. MCMV Infection-Induced Myocardial Remodeling in C57BL/6 Mice

**Figure 2:**
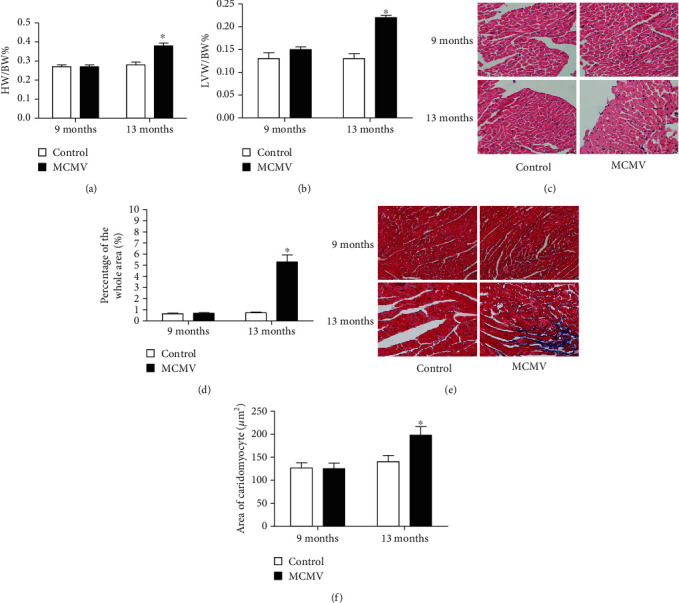
MCMV infection induced myocardial remodeling in mice. (a) Determination of HW/BW%. (b) Determination of LVW/BW%. (c) Representative images of H&E staining in the heart. (d) Quantitative analysis of the cell size (*μ*m^2^) of cardiomyocytes in all groups. (e) Representative images of Masson's trichrome stain in the heart. (f) Quantitative analysis of fibrosis in all groups. The data are expressed as the means ± SEM (*n* = 5), ^∗^*P* < 0.05, the MCMV groups vs. the age-matched control groups. HW/BW%: heart-to-body weight ratio; LVW/BW%: left ventricle-to-body weight ratio. Images were magnified to ×400 power, scale bars = 20 *μ*m.

Downregulation of miR-1929-3p and the increase in BP observed in infected mice, which were consistent with the results of previous studies, prompted us to assess whether there were subsequent structural changes in target organs. HW/BW% and LVW/BW% were calculated to reflect the size of the entire heart and to evaluate left ventricular hypertrophy, respectively. Both ratios were significantly higher in MCMV-infected than in control mice at the age of 13 months (*P* < 0.05, Figures 2(a) and 2(b)). Morphologically, as shown in Figures 2(c) and 2(d), H&E staining revealed a larger cross-sectional area of cardiomyocytes in the MCMV group compared to the control group at 13 months of age (*P* < 0.05). Similarly, Masson's trichrome staining showed an increase in interstitial fibrin and collagen in the MCMV group at the same age (*P* < 0.05, Figures 2(e) and 2(f)).

### 3.3. miR-1929-3p Overexpression Improves Myocardial Remodeling in C57BL/6 Mice

**Figure 3:**
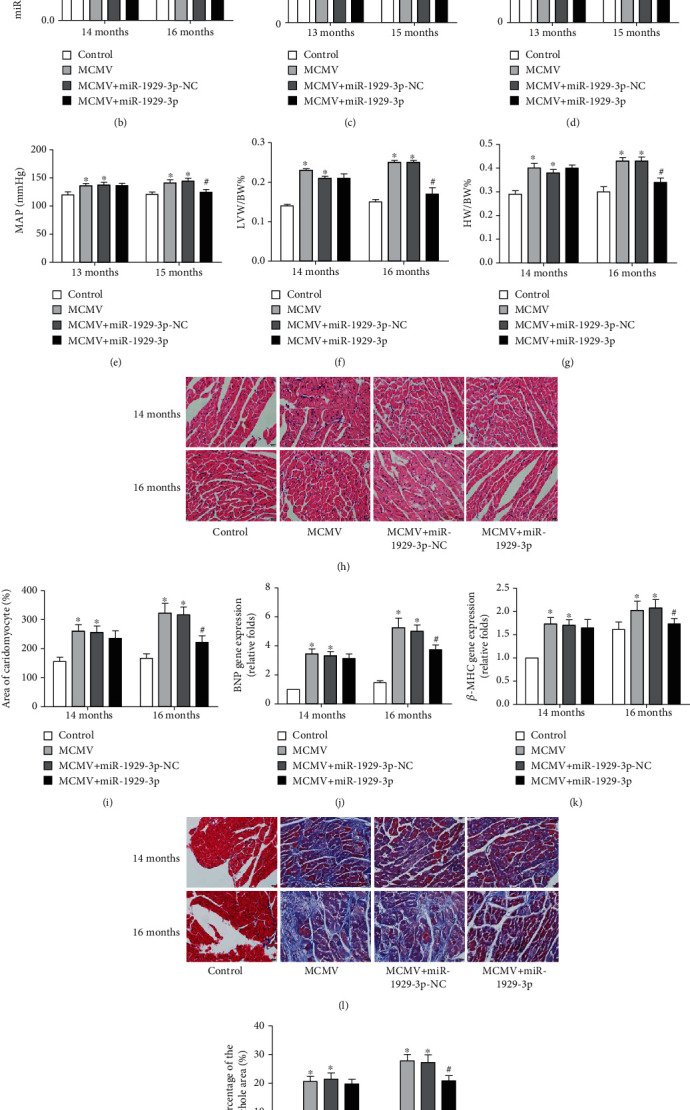
miR-1929-3p overexpression relieved MCMV-induced myocardial remodeling in mice. (a) Schematic of intervention here and in Figures 4–7 in the experimental group. (b) The expression of miR-1929-3p in the myocardium. The value was normalized to the respective control groups, which was set to 1: (c) SBP; (d) DBP; (e) MAP; (f) HW/BW%; (g) LVW/BW%. (h) Representative images of H&E staining. (i) Quantitative analysis of the cell size (*μ*m^2^) of cardiomyocytes in all groups. (j, k) qRT-PCR analysis of cardiac-specific fetal genes BNP and *β*-MHC. The value was normalized to the respective control groups, which was set to 1. (l) Representative images of Masson's trichrome stain. (m) Quantitative analysis of fibrosis in all groups. The data are expressed as the means ± SEM (*n* = 5), ^∗^*P* < 0.05 vs. the age-matched control groups. ^#^*P* < 0.05, the MCMV+miR-1929-3p groups vs. the age-matched MCMV groups. Images were magnified to ×400 power; scale bars = 20 *μ*m.

From the above experiments, we concluded that myocardial remodeling occurred in 13-month-old mice after 6 months (from 7 to 13 months of age) of MCMV infection, accompanied by a decrease in miR-1929-3p expression. In a subsequent experiment, we injected rAAV-miR-1929-3p overexpression vector in mice through the tail vein at 13 months of age (Figure 3(a)). qRT-PCR results showed that, upon overexpression, miR-1929-3p levels significantly increased in mice at both 14 and 16 months (*P* < 0.05, Figure 3(b)). Remarkably, miR-1929-3p overexpression relieved the increase in SBP, DBP, and MAP in MCMV-infected mice at 15 months of age (*P* < 0.05, Figures 3(c)–3(e)). In terms of myocardial remodeling, the average HW/BW% and LVW/BW% increased at various time points in the MCMV group. Interestingly, both HW/BW% and LVW/BW% were reduced in the MCMV+miR-1929-3p group at 16 months of age (*P* < 0.05, Figures 3(f) and 3(g)). Morphological observation of H&E staining revealed a continuously increased cross-sectional area of cardiomyocytes in the MCMV group (*P* < 0.05), which was alleviated in the MCMV+miR-1929-3p group at 16 months of age (*P* < 0.05, Figures 3(h) and 3(i)). Next, we determined the mRNA expression of cardiac hypertrophy-related genes. Expression of BNP and *β*-MHC was markedly higher in the MCMV group than in the control group (*P* < 0.05), but again decreased in the MCMV+miR-1929-3p group at 16 months of age (*P* < 0.05, Figures 3(j) and 3(k)). Consistently, Masson's trichrome staining showed an increase in interstitial fibrin and collagen in the MCMV group at 14 and 16 months (*P* < 0.05), which was attenuated following miR-1929-3p overexpression at 13 months of age (*P* < 0.05, Figures 3(l) and 3(m)).

### 3.4. Regulation of Ednra by Overexpressed miR-1929-3p Alleviates Cardiac Dysfunction Induced by MCMV Infection

**Figure 4:**
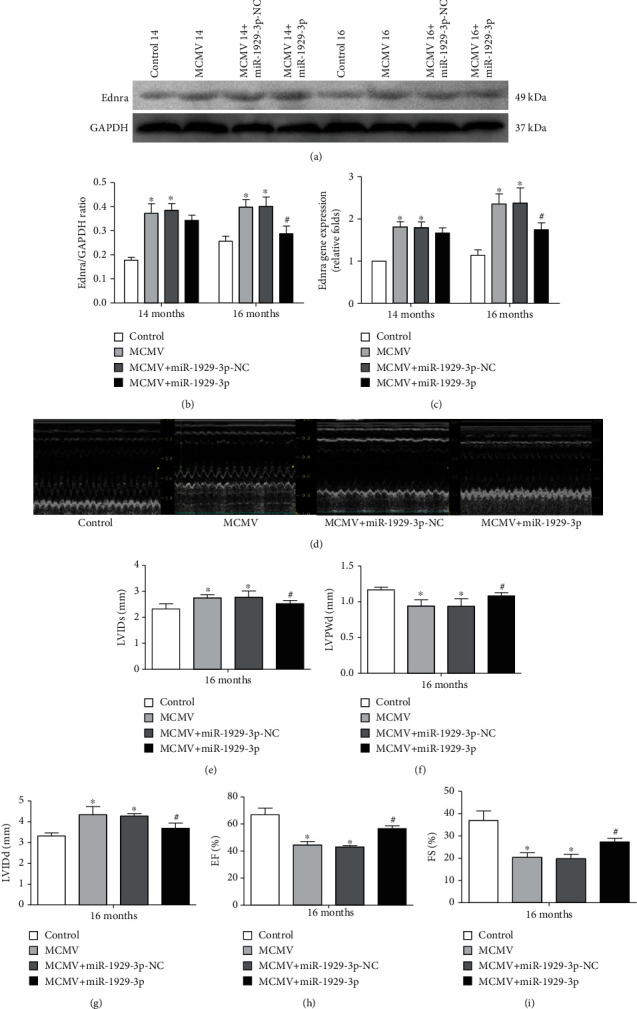
miR-1929-3p overexpression downregulated Ednra and alleviated MCMV-induced cardiac dysfunction. (a) Western blotting analysis of Ednra protein. (b) Densitometric analysis of (a). (c) qRT-PCR analysis of Ednra mRNA. The value was normalized to the control groups, which was set to 1. (d) Images of the M-mode of LV. (e–i) LVPWd, LVIDs, LVIDd, EF%, and FS%. The data are expressed as the means ± SEM (*n* = 5), ^∗^*P* < 0.05 vs. the age-matched control groups. ^#^*P* < 0.05, the MCMV+miR-1929-3p groups vs. the MCMV groups. LVPWd: left ventricular end-diastolic posterior wall dimension; LVIDs: left ventricular end-systolic diameter; LVIDd: left ventricular end-diastolic diameter; EF%: ejection fraction; FS%: fractional shortening.

As suggested by our previous studies, Ednra is targeted and negatively regulated by miR-1929-3p. Consistent with these results, western blot (Figures 4(a) and 4(b)) and qRT-PCR (Figure 4(c)) analysis of mouse cardiac muscle indicated that the protein and mRNA expression of Ednra significantly increased in the MCMV group at both 14 and 16 months of age (*P* < 0.05). However, this tendency was reversed at 16 months in the MCMV+miR-1929-3p group (*P* < 0.05). Next, to investigate the effect of improved blood pressure and myocardial remodeling on cardiac function, we performed in vivo echocardiographic measurements in 16-month-old mice (Figure 4(d)). We observed distinctly reduced LVPWd, EF%, and FS%, along with significantly increased left ventricular internal diameter end diastole and end systole (LVIDs and LVIDd) in the MCMV group (*P* < 0.05, Figures 4(e)–4(i)), indicating that cardiac function was significantly affected in infected animals, consistent with myocardial remodeling. However, 3 months after miR-1929-3p overexpression, cardiac function improved to varying extent (*P* < 0.05, Figures 4(d)–4(i)).

### 3.5. miR-1929-3p Overexpression Suppresses the Expression of NLRP3 Inflammasome in the Myocardium of C57BL/6 Mice

**Figure 5:**
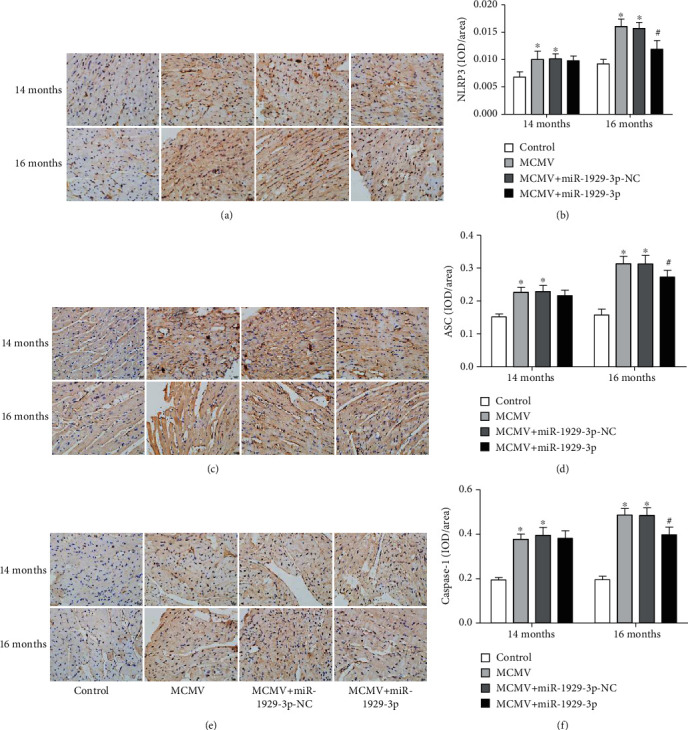
miR-1929-3p overexpression reduced MCMV-induced expression of the NLRP3 inflammasome of the myocardium in morphology. (a, c, e) Immunohistochemical analysis of NLRP3, ASC, and caspase-1. (b, d, f) Densitometric analysis of (a), (c), and (e). The data are expressed as the means ± SEM (*n* = 5), ^∗^*P* < 0.05 vs. the age-matched control groups. ^#^*P* < 0.05, the MCMV+miR-1929-3p groups vs. the age-matched MCMV groups. Images were magnified to ×400 power; scale bars = 20 *μ*m.

**Figure 6:**
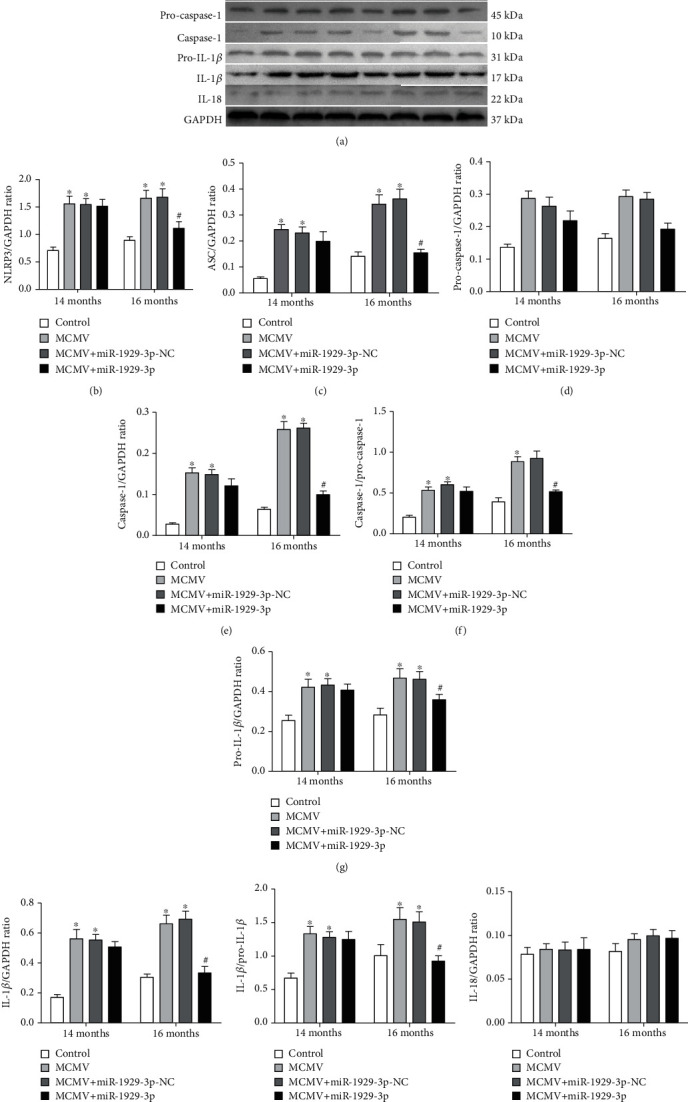
miR-1929-3p overexpression suppressed MCMV-induced expression of the NLRP3 inflammasome and secretion of IL-1*β* in the myocardium after MCMV infection. (a) Western blotting analysis of NLRP3, ASC, pro-caspase-1, caspase-1, pro-IL-1*β*, IL-1*β*, and IL-18 protein. (b–j) Densitometric analysis of (a). The data are expressed as the means ± SEM (*n* = 5), ^∗^*P* < 0.05 vs. the age-matched control groups. ^#^*P* < 0.05, the MCMV+miR-1929-3p groups vs. the age-matched MCMV groups.

Next, we detected the expression of NLRP3 inflammasome components to determine the activation of this complex in our model. Immunohistochemistry of mouse cardiac tissues showed higher expression of NLRP3, ASC, and caspase-1 in the MCMV group than in the control group; however, their expression was significantly lower in the MCMV+miR-1929-3p group than in the MCMV group at 16 months of age (*P* < 0.05, Figures 5(a)–5(f)). Western blot analysis (Figures 6(a)–6(j)) showed that expression of NLRP3, ASC, caspase-1, and IL-1*β*, as well as the levels of activated caspase-1 and IL-1*β*, were significantly increased in the MCMV group (*P* < 0.05). However, the levels of IL-18 did not change significantly (*P* > 0.05). Subsequently, these changes declined after 3 months of miR-1929-3p overexpression (*P* < 0.05).

### 3.6. miR-1929-3p Overexpression Reduces ET-1 and IL-1*β* Activation in C57BL/6 Mice

**Figure 7:**
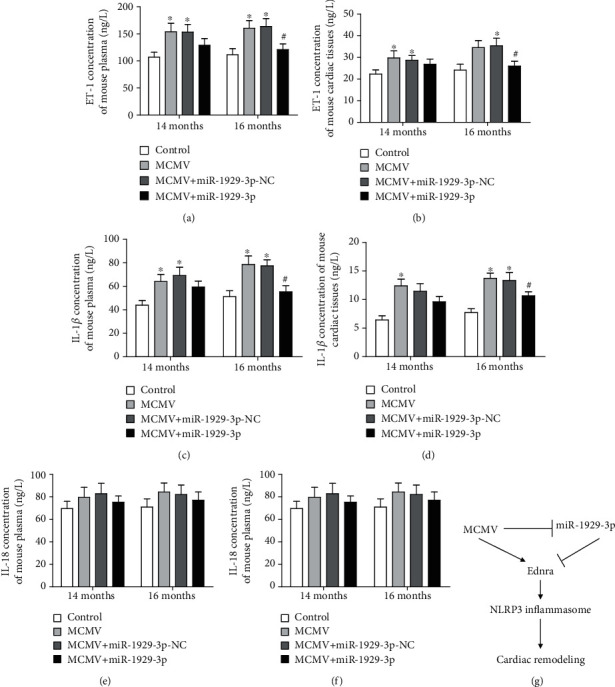
miR-1929-3p overexpression decreased ET-1 and IL-1*β* activation in MCMV-infected mice. (a, c, e) ELISA detection of ET-1, IL-1*β*, and IL-18 concentration in plasma. (b, d, f) ELISA detection of ET-1, IL-1*β*, and IL-18 concentration in cardiac tissues. The data are expressed as the means ± SEM (*n* = 5), ^∗^*P* < 0.05 vs. the age-matched control groups. ^#^*P* < 0.05, the MCMV+miR-1929-3p groups vs. the age-matched MCMV groups. (g) A schematic model showing the essential role of miR-1929-3p and Ednra in MCMV-induced hypertensive cardiac remodeling.

Therefore, we analyzed the levels of ET-1, IL-1*β*, and IL-18 in the plasma and myocardium of infected mice by ELISA. The levels of ET-1 and IL-1*β* were both significantly increased in the plasma and myocardia of MCMV-group animals (*P* < 0.05, Figures 7(a)–7(d)), while those of IL-18 were only slightly changed (*P* > 0.05, Figures 7(e) and 7(f)). However, injection of the miR-1929-3p overexpression vector significantly decreased ET-1 and IL-1*β* expression in the myocardium (*P* < 0.05, Figures 7(a)–7(d)).

## 4. Discussion

We explored the possible mechanism connecting CMV infection with hypertension and myocardial remodeling through miR-1929-3p. Carotid invasive BP measurement showed a significant increase of BP in the MCMV group, consistent with the results of Cheng et al. [21] The increased HW/BW% and LVW/BW% in 13-month-old MCMV-infected mice and H&E and Masson's staining revealed hypertrophy and fibrosis of cardiomyocytes in progress. Collectively, the data shows the successful establishment of the hypertensive cardiac hypertrophy model. In this study, we measured the expression of miR-1929-3p specifically in the myocardium. The results showed obvious downregulated miR-1929-3p expression and significantly increased BP in the early stage after MCMV infection. Then, remarkably aggravated cardiac hypertrophy and fibrosis appeared in the advanced stage. This is consistent with the progression of EH.

HCMV infection has been recently associated with the development of various conditions, including tumors, atherosclerotic cardiovascular diseases, and Alzheimer's disease, etc., in which there is no clear evidence of viral replication [4]. Previous research by our team has shown that HCMV is one of the most important factors promoting EH development, through inflammation, oxidative stress, renin-angiotensin-aldosterone system (RAAS) activation, and interaction with related environmental risk factors that may increase EH susceptibility [28–31]. Following infection, to replicate steadily in the host, HCMV has the potential to express functional miRNAs related to viral replication and intracellular transcription, resulting in changes in host miRNA expression, and facilitating viral infection or persistent latent infection by avoiding targeted intracellular miRNA degradation, immune escape, inhibition of apoptosis, promotion of cell growth, or other mechanisms [13, 32]. Therefore, the mechanism by which HCMV infection leads to hypertensive heart disease may be related to both host- and virus-encoded miRNAs. Here, we found that the host-encoded miR-1929-3p is distinctly downregulated, accompanied by increased BP and adverse cardiac remodeling. Therefore, we overexpressed miR-1929-3p to confirm its role in the progression of this cardiovascular disease and to explore the possible pathogenic mechanism and associated signaling pathway.

CMV has strict species specificity for infection, which precludes the establishment of infected experimental animals with HCMV. MCMV infection has been the most suitable model to understand HCMV-associated disease and to answer basic questions that cannot easily be addressed in clinical research so far [4]. In this study, although the levels of IgM and IgG antibodies in the MCMV-infected mice increased at 9 months of age, myocardial remodeling was not obvious until 13 months of age. We speculate that this might be because IgM and IgG concentrations did not reach the critical value of organ damage in the early stage of infection. On the other hand, the process leading from hypertension to visible cardiac remodeling is chronic, which explains these experimental results.

In this study, we constructed a miR-1929-3p overexpression vector coated with rAAV, which is considered as an ideal carrier for gene therapy because of low immunogenicity, nonpathogenicity, long-term expression, and serotype-dependent tissue affinity [33]. Boureima et al. found that type 9 rAAV can steadily infect the myocardium of C57BL/6 mice [34]. In this study, miR-1929-3p overexpression applied at the age of 13 months (when distinct myocardial remodeling was revealed). Echocardiography indicated that the mice selected in our study were just in the decompensated period of hypertrophy. However, overexpression of miR-1929-3p markedly ameliorated cardiac function in mice. Consistently, the decrease index including HW/BW%, LVW/BW%, and cardiomyocyte cross-sectional area also revealed improved ventricular remodeling. Undoubtedly, overexpression of miR-1929-3p exerts a protective effect on hypertensive myocardial structure and dysfunction induced by MCMV infection.

In patients with hypertension, Ednra can increase vasoconstrictive tension, which may be related to the increase in ET-1 [35], and the use of endothelin receptor antagonists can significantly improve the target organ damage caused by EH [36]. In this study, we found that the levels of Ednra were significantly increased in the myocardial tissue of MCMV-infected mice, but significantly decreased with miR-1929-3p overexpression, suggesting that miR-1929-3p may influence remodeling of the hypertensive myocardium induced by MCMV infection through its target gene Ednra. Chen et al. demonstrated that kefir peptides can exert anti-inflammatory and antifibrotic effects on target organs of spontaneous hypertension by simultaneously reducing the expression of ET-1 and NLRP3 [37]. Bao et al. found that Coxsackie virus B3 can activate inflammatory bodies and induce viral myocarditis [38]. Therefore, we hypothesized that both Ednra and NLRP3 may participate in MCMV-induced myocardial remodeling and discovered caspase-1 shearing and IL-1*β* release initiated by NLRP3 activation, as well as increased ET-1 level after MCMV infection, which were reduced following miR-1929-3p overexpression. Interestingly, IL-18 levels in the plasma and myocardium did not markedly change. Schmidt et al. showed that in the absence of ROS, host cells of C57BL/6 mice selectively produce IL-18 but not IL-1*β* in response to NLRP3 inflammasome activation induced by *Listeria monocytogenes* p60 protein, revealing that distinct cofactors license NLRP3 inflammasomes for induction of downstream responses, and can independently govern processing and secretion of IL-1*β* and IL-18 [39]. In addition, IL-1*β* has been ascribed a probacterial role in some bacterial infection models [40]. Based on this evidence, we speculate that MCMV may promote its survival and DNA replication in the host by selectively triggering the secretion of IL-1*β*.

One of the limitations of our research is that, due to the species specificity of CMV infection, we used an animal model of hypertension, which can only partially represent the pathophysiological changes occurring in human hypertension. In addition, as the effects of CMV on miRNA expression in humans and mice might be different, further research and adequate evidence are required. Moreover, miRNAs are vulnerable to several factors and can also be involved in the regulatory networks of disease-related pathways. Considering that miR-1929-3p has been scarcely reported in the literature, we cannot determine whether MCMV specifically leads to the low expression of miR-1929-3p and subsequent hypertension and adverse myocardial remodeling; whether other pathogens can influence the occurrence and development of cardiovascular diseases through regulation of this miRNA remains to be further clarified. Finally, the effects of miR-1929-3p on MCMV-induced cardiac remodeling and changes in NLRP3 inflammasome activity require further analysis and verification in vitro and at the molecular level.

CMV is closely related to several cardiovascular diseases, although the specific underlying mechanism has not been elucidated. Our findings document the protective functions of miR-1929-3p overexpression in hypertension and subsequent myocardium remodeling induced by MCMV infection. As one of the receptors of ET-1, Ednra exerts adverse effects on myocardial remodeling through the RAAS system [41]. We demonstrated that MCMV infection may be another mechanism of Ednra activation, promoting hypertension-related myocardial remodeling through cascade activation of the NLRP3 inflammasome. Notably, overexpression of miR-1929-3p may effectively suppress EH myocardial remodeling by inhibiting expression of Ednra. Treatment of HCMV infections with currently available drugs targeting viral enzymes is often limited by severe side effects and emergence of drug-resistant viruses [42]. Therefore, our study provides new basic research evidence to improve the diagnosis and treatment of CMV-induced cardiovascular disease. Although there are numerous difficulties in drug development of small-molecule agents, miR-1929-3p or its biologicals are promising candidates for future clinical application in the treatment of EH cardiac dysfunction.

## 5. Conclusion

In summary, we found that MCMV infection decreased the expression of miR-1929-3p, which increased the expression of the target gene Ednra. In turn, Ednra promoted the activation of the NLRP3 inflammasome and the maturation and release of IL-1*β*, finally leading to EH myocardial remodeling. Our crucial finding is that the change in the Ednra/NLRP3 axis, induced by miR-1929-3p downregulation, may be involved in MCMV-induced hypertensive myocardial remodeling. Intervention with the rAAV-miR-1929-3p overexpression vector, an innovation in this study, significantly relieved hypertensive myocardial remodeling and dysfunction induced by viral infection, playing an undeniable therapeutic effect.

## Data Availability

The data used to support the findings of this study are available from the corresponding authors upon request.
